# Optimizing Care for Undescended Testicles in Children and Adolescents—Diagnosis, Management, and Outcomes: A Narrative Review of Current Evidence

**DOI:** 10.3390/children13050633

**Published:** 2026-05-01

**Authors:** Marko Bašković, Jana Buzuk, Bianka Dujić, Danijela Jurić, Kristina Jurković, Karla Pehar, Sara Vuković, Davor Ježek, Dubravko Habek, Ivan Milas

**Affiliations:** 1 School of Medicine, Catholic University of Croatia, Ilica 242, 10000 Zagreb, Croatia; 2 Department of Pediatric Surgery, Children’s Hospital Zagreb, Ulica Vjekoslava Klaića 16, 10000 Zagreb, Croatia; 3 School of Medicine, University of Zagreb, Šalata 3, 10000 Zagreb, Croatia; 4 Scientific Centre of Excellence for Reproductive and Regenerative Medicine, School of Medicine, University of Zagreb, Šalata 3, 10000 Zagreb, Croatia; 5 Croatian Academy of Medical Sciences, Kaptol 15, 10000 Zagreb, Croatia; 6 Department of Histology and Embryology, School of Medicine, University of Zagreb, Šalata 3, 10000 Zagreb, Croatia; 7 Department of Transfusion Medicine and Transplantation Biology, University Hospital Centre Zagreb, Kišpatićeva 12, 10000 Zagreb, Croatia; 8 Department of Obstetrics and Gynecology, Clinical Hospital Merkur, Zajčeva ulica 19, 10000 Zagreb, Croatia; 9 Department of Surgical Oncology, University Hospital for Tumors, Sestre milosrdnice University Hospital Center, Ilica 197, 10000 Zagreb, Croatia

**Keywords:** cryptorchidism, undescended testis, orchidopexy, subfertility, testicular cancer, children, adolescents, pediatric surgery, pediatric urology

## Abstract

Cryptorchidism is the most prevalent congenital anomaly of the male genitourinary tract, with an incidence of approximately 1 to 9 percent in full-term male infants, decreasing with age due to spontaneous descent. It encompasses testes that fail to descend into the scrotum, which may be intra-abdominal, inguinal, or ectopic, and can be associated with syndromic, genetic, or environmental factors. The descent process occurs in two phases: intra-abdominal, driven by gubernacular development and androgen-independent mechanisms, and inguinoscrotal, regulated by hormonal and mechanical factors including androgens and the gubernaculum. Clinically, cryptorchidism manifests as absent or hypoplastic scrotal testes, often with inguinal fullness. Palpation and physical examination are primary diagnostic tools, with imaging such as ultrasound or MRI reserved for specific cases. Surgical exploration remains the definitive diagnostic modality, especially for nonpalpable testes. Early referral, ideally before 12 months of age, is essential for timely orchidopexy, which aims to position the testes within the scrotum to reduce risks of torsion, trauma, subfertility, and malignancy. Hormonal therapy shows limited efficacy and is generally not recommended as a primary treatment modality. Long-term outcomes indicate that early orchidopexy improves spermatogenic potential and fertility. Men with a history of cryptorchidism exhibit elevated risks of subfertility and testicular germ cell tumors, with the risk being higher if surgical correction is delayed or if testes remain intra-abdominal. The increased malignancy risk persists even after orchidopexy, underscoring the importance of vigilant surveillance. Management strategies emphasize a multidisciplinary approach, combining surgical intervention with ongoing monitoring, to optimize functional and oncological outcomes. Early diagnosis, appropriate surgical treatment, and patient education are critical components in minimizing long-term complications associated with cryptorchidism.

## 1. Introduction

Cryptorchidism, also known as undescended testis, is the most prevalent congenital anomaly of the male genitourinary tract, with an incidence of approximately 1–9% in full-term male infants, decreasing to about 1% by the age of one year due to spontaneous descent [1,2]. This condition encompasses a spectrum of testicular maldescent, which includes testes that are absent or located outside the scrotum, either due to failure of descent or postnatal ascent [3]. Cryptorchidism refers to a testis that is not within the scrotal sac and has not descended spontaneously into the scrotum by four months of age (or corrected gestational age in preterm infants). The term implies a “hidden testis,” which may either be present but undescended or absent due to agenesis or atrophic processes [4,5]. Classification of undescended testes can be systematically approached by first distinguishing between palpable and non-palpable testes. Palpable testes are those that can be felt on physical examination, typically located within the inguinal canal or just outside the external inguinal ring. Non-palpable testes, on the other hand, are not felt on examination and may be intra-abdominal, absent, or atrophic. This initial classification guides subsequent diagnostic and management strategies, with palpable testes often amenable to surgical correction via inguinal approaches, and non-palpable testes requiring imaging and potentially laparoscopy for localization [1,2,3,4,5,6].

Absent testis can result from agenesis, where the testis fails to develop, or from intrauterine vascular insults leading to atrophy, such as prenatal testicular torsion. This condition is often termed vanishing testis syndrome or testicular regression syndrome and is characterized by the absence of testicular tissue on imaging and surgical exploration [2,6]. Boys with bilateral absence of testes are diagnosed with anorchia [7]. Undescended testes are those that halt along their normal descent pathway, which begins intra-abdominally and proceeds through the inguinal canal to the scrotum. These testes may be intra-abdominal, palpable within the inguinal canal (intracanalicular), or just outside the external inguinal ring (suprascrotal) [2,8]. Retractile testes are normally descended testes that are hyperresponsive to cremasteric reflexes, allowing them to be pulled into a suprascrotal position. These testes can be brought into a dependent scrotal position and typically remain there unless the cremasteric reflex is stimulated again [9,10]. Ascending testes describe testes that are initially located within the scrotum during early childhood but subsequently “ascend” or migrate proximally, resulting in acquired undescended testes before puberty. The precise age of ascent varies, and this phenomenon is increasingly recognized as an acquired form of cryptorchidism [11,12]. Ectopic testes descend normally through the external inguinal ring but deviate from their typical pathway to settle in aberrant locations such as the suprapubic region, femoral canal, perineum, superficial inguinal pouch (most common site), or contralateral scrotal compartment (least common). Noninguinal ectopic testes account for less than 1% of undescended testes [13].

The process of testicular descent occurs in two primary phases: the intra-abdominal phase and the inguinoscrotal phase. While the mechanisms governing normal descent are not fully elucidated, it is understood that the intra-abdominal phase, which occurs between weeks 8 and 15 of gestation, is largely androgen-independent and mediated by the gubernaculum, which guides the testis toward the inguinal canal. The subsequent inguinoscrotal phase begins around the 28th week of gestation and involves complex hormonal, mechanical, and neurochemical interactions. Key factors include androgen signaling, gubernacular development, and the patency of the processus vaginalis [14,15,16]. Experimental and clinical studies suggest roles for mechanical forces such as intra-abdominal pressure, hormonal mediators like androgens, Müllerian inhibiting substance (MIS), calcitonin gene-related peptide, and processes involving gubernacular regression [17,18].

The etiology of cryptorchidism is multifactorial. Abnormalities in hormonal signaling—such as gonadotropin deficiency, reduced MIS levels, or excess placental estrogen production—may interfere with normal descent. Genetic factors, environmental influences, and intrauterine vascular events like prenatal torsion may also contribute. The pathogenesis of absent testes often involves prenatal vascular compromise, leading to testicular atrophy or agenesis [5,19,20]. Conversely, ascending testes are hypothesized to result from laxity in the spermatic cord structures, allowing the testes to initially descend into the scrotum and subsequently become tethered or pulled out during growth [21,22].

This narrative review aims to synthesize the current evidence, guidelines, and clinical practices related to the diagnosis, management, and outcomes of undescended testicles in children and adolescents. Its contribution lies in providing an up-to-date, comprehensive, and integrated overview to inform clinical decision-making, improve long-term reproductive and oncological outcomes, and identify areas for future research.

## 2. Epidemiology, Risk Factors, and Associated Conditions

Geographical variations in the prevalence of cryptorchidism have been documented [23]. For instance, a prospective cohort study reported a prevalence of approximately 9% in newborn boys in Denmark, compared to only 2.4% in Finland [24]. These disparities may result from genetic differences, environmental exposures (such as endocrine-disrupting chemicals and lifestyle factors), or variations in clinical definitions—particularly distinctions between truly undescended versus retractile testes.

Several maternal, fetal, and environmental factors have been associated with increased risk. Prematurity and low birth weight (less than 2.5 kg) are well-established risk factors. Infants born small for gestational age also demonstrate increased susceptibility, suggesting intrauterine growth restriction may influence testicular descent [25,26,27]. Prenatal exposure to endocrine-disrupting chemicals such as diethylstilbestrol, pesticides, and phthalates has been linked to cryptorchidism in some epidemiological studies. These agents may interfere with hormonal pathways critical for testicular descent [19].

While cryptorchidism is predominantly an isolated anomaly, it can be associated with various congenital syndromes, genetic disorders, and anatomical abnormalities, especially in bilateral cases. Associations include abdominal wall defects like prune belly syndrome (Eagle-Barrett syndrome), neural tube defects such as myelomeningocele, and neurological conditions like cerebral palsy. Conditions involving differences in sex development (DSD), including mixed gonadal dysgenesis, ovotesticular DSD, and persistent Müllerian duct syndrome, have also been linked to cryptorchidism; notably, the coexistence of cryptorchidism and hypospadias increases the likelihood of an underlying DSD [28,29,30,31]. Furthermore, genetic syndromes affecting endocrine function, such as Kallmann syndrome, Klinefelter syndrome, and Prader–Willi syndrome, have been associated with cryptorchidism. These syndromes often involve diminished testosterone secretion. Other genetic disorders impacting gonadotropin or testosterone action, including androgen insensitivity syndrome, are also linked. Additionally, chromosomal abnormalities such as trisomy 18, trisomy 13, 22q11.2 deletion syndrome, 1p36 deletion syndrome, Beckwith-Wiedemann syndrome, Smith-Lemli-Opitz syndrome, and Cornelia de Lange syndrome have been associated with cryptorchidism, although their occurrence is less frequent [32,33,34].

Genetic contributions to cryptorchidism are complex and multifactorial. While genetic alterations are relatively uncommon, they are more frequent than in the general population. For example, a case–control study identified genetic abnormalities in 17 of 600 boys with cryptorchidism (2.8%) compared to only 1 of 300 boys without the condition (0.3%). The genetic alterations in affected boys included Klinefelter syndrome and mutations affecting the insulin-like factor 3 (INSL3) pathway, such as mutations in the RXFP2 receptor gene. Additional studies have reported mutations in the INSL3 gene itself and the androgen receptor gene, emphasizing the critical role of hormonal signaling pathways in testicular descent [32,33,35,36].

## 3. Clinical Features and Evaluation

Cryptorchidism typically presents with an absent or hypoplastic scrotum that often exhibits poor rugation or asymmetry [37]. Palpable inguinal fullness may be observed due to the presence of the undescended testis or associated hydrocele or hernia sac. Approximately 10% of cryptorchidism cases are bilateral, with bilateral involvement more frequently associated with underlying syndromic or genetic conditions. Among cases where the testis cannot be palpated unilaterally, there is a tendency for the left side to be more commonly affected [2,5,38].

The most common anatomical location for an undescended testis is just outside the external inguinal ring (suprascrotal or prepubic position), followed by intracanalicular (within the inguinal canal), and ultimately intra-abdominal positions. Nonpalpability of at least one testis occurs in approximately 20% of boys with cryptorchidism, often correlating with intra-abdominal positioning [39,40,41]. Most testes that are undescended at birth complete their descent within the first four months of life, driven by hormonal and anatomical factors. Failure to descend by this age suggests a low likelihood of spontaneous descent, and surgical intervention—orchiopexy—is generally indicated [42]. Conversely, some testes that descend during infancy may later ascend, a condition known as ascending or acquired cryptorchidism, typically occurring between ages four and eight. Such cases often require surgical correction to preserve testicular function [12,22].

True undescended testes are associated with several potential complications and sequelae, including inguinal hernia, increased risk of testicular torsion, trauma—particularly of intracanalicular testes due to compression against the pubic bone—subfertility, and an increased risk of malignant transformation [5,43]. Ectopic testes, which are located outside the normal path of descent, may also be prone to trauma and impaired spermatogenesis. Early recognition of cryptorchidism by primary care providers and timely referral for specialized management are crucial in minimizing these risks. Prompt intervention has been shown to significantly reduce the likelihood of adverse outcomes such as infertility and testicular cancer [44].

A thorough clinical history is essential and should include details about the position of the testes during the neonatal period, as testes in the scrotum at that time are more likely to be retractile rather than truly undescended. Previous inguinal or scrotal surgeries should be noted, as these may indicate iatrogenic cryptorchidism resulting from scar tissue tethering. It is also important to inquire about evidence of endocrine abnormalities during pregnancy, such as maternal androgen exposure, which can influence fetal genital development, and to gather information about family history concerning unexplained neonatal deaths, genital anomalies, pubertal disorders, or infertility, as these may suggest underlying genetic or endocrinologic conditions [45,46,47].

The physical examination should be comprehensive. While most cases of cryptorchidism are isolated, the general examination should include assessment for features suggestive of syndromic or metabolic disorders, such as dysmorphic features, midline defects like nystagmus, hypertelorism, cleft palate, or other skeletal anomalies [48]. The genital examination requires a systematic approach; palpation of the testes and inguinal region should be performed with two hands, starting from the anterior superior iliac spine and moving along the inguinal canal toward the scrotum. Techniques such as warming the inguinal area or positioning the child in cross-legged, squatting, or knee-chest positions can facilitate detection. To differentiate retractile from truly undescended or ectopic testes, maneuvers involving sustained elevation of the testis into the dependent scrotal position are useful; retractile testes tend to remain in the scrotum after such maneuvers, while ectopic testes typically migrate out of the scrotum. A maneuver where the cremasteric reflex is fatigued by holding the testis in the dependent position for at least one minute can help distinguish these conditions. If uncertainty persists, consultation with a pediatric urologist is advised [49,50,51,52].

When a testis is palpable, its position, size, and consistency should be documented, comparing with the contralateral testis. Palpable residual tissue, such as a nubbin, may be atrophic testicular tissue, but it can also represent gubernaculum or attached epididymis and vas deferens, which are not specific enough to exclude intra-abdominal testis [52,53]. Therefore, surgical exploration remains the gold standard for definitive diagnosis when intra-abdominal or nonpalpable testes are suspected [54,55].

In cases where testes are nonpalpable in the inguinal canal, scrotum, or typical ectopic sites, further evaluation with imaging modalities such as ultrasound, MRI, or diagnostic laparoscopy is indicated to localize intra-abdominal testes or confirm absence [54,56]. The presence of a unilateral nonpalpable testis with contralateral testicular hypertrophy can suggest testicular absence or atrophy, particularly after puberty; however, this finding alone is insufficient to exclude intra-abdominal testis, and surgical exploration is often necessary [57]. Examination of external genitalia should include assessment of the phallus size, urethral meatus position, and scrotal morphology. A small phallus may indicate hypogonadism or disorders of sex development (DSD), while hypospadias, especially with scrotal or perineal meatus, may be associated with DSDs. A hypoplastic or poorly rugated scrotum, or a bifid scrotum, can also suggest abnormal genital development [58,59]. The inguinal area should be carefully examined for fullness, which may indicate an inguinal hernia or the presence of a testis within the canal. Since a patent processus vaginalis is present in approximately 90% of cases of undescended testes, inguinal hernias are common and may occur at any time, emphasizing the importance of surgical correction to prevent incarceration [60].

## 4. Imaging

Routine imaging is generally not indicated for locating nonpalpable testes, as it has limited diagnostic utility and does not reliably influence management decisions. Specifically, imaging modalities such as ultrasonography lack sufficient sensitivity and specificity to replace surgical exploration, which remains the definitive diagnostic approach. A systematic review reported that ultrasonography has a sensitivity of approximately 45% and a specificity of 78% for detecting nonpalpable testes, indicating that it misses a significant proportion of cases and produces false positives [61,62]. In contrast, diagnostic surgical exploration approaches near 100% accuracy for locating undescended testes and evaluating their viability [63]. Accordingly, imaging should be reserved for specific clinical scenarios rather than employed as a routine step.

Situations where imaging may be beneficial include assessment for disorders of sexual development (DSD), where ultrasonography can be used to evaluate for the presence of gonads or a uterus in phenotypically male infants with bilateral nonpalpable testes, aiding in the diagnosis of DSD variants [64]. Additionally, ultrasonography may assist in preoperative planning in obese patients, where intra-abdominal or intracanalicular testes may be difficult to palpate. Identifying the testis location through imaging in such cases can influence the surgical approach—potentially shifting from a laparoscopic to an inguinal procedure [65].

It is noteworthy that a systematic review assessing the accuracy of ultrasonography in localizing inguinal or scrotal testes reported a pooled accuracy of approximately 92%. However, the studies included were of variable quality, often lacking important clinical details such as the position of the contralateral testis or associated anomalies, which limits the generalizability of these findings. In summary, while imaging can be useful in select cases, it should not replace surgical exploration for definitive diagnosis and management of nonpalpable testes [61,66].

## 5. Diagnostic Approach and Timely Referral

The evaluation of cryptorchidism fundamentally depends on whether the affected testis is palpable or nonpalpable. Accurate diagnosis is crucial for guiding management strategies and predicting outcomes. In cases where the testis is palpable, the primary differential diagnoses include true undescended testis, retractile testis, and ectopic testis. Differentiation typically relies on a thorough history and physical examination, although ambiguous cases may necessitate referral to a pediatric urologist for further assessment [27,37].

A true undescended testis is characterized by a testis palpable along the normal descent pathway—most often within the inguinal canal (intracanalicular) or just external to the external inguinal ring (suprascrotal) [67]. Clinically, such testes may “slide” under gentle pressure during examination, indicating a fixed position since birth. Imaging modalities generally have limited utility in confirming the diagnosis; instead, clinical examination remains the gold standard. A retractile testis usually can be manipulated into the dependent scrotal position and will remain there if the cremasteric reflex is suppressed, such as by manual stabilization. Differentiating retractile from true undescended testes can be challenging, as retractile testes may ascend spontaneously during activity. Persistent retractile testes often require observation, but if uncertainty persists, referral to a pediatric urologist is recommended for further evaluation, including dynamic testing. Ectopic testes are located outside the normal path of descent, commonly found in areas such as the perineum, femoral canal, suprapubic region, or superficial inguinal pouch. These testes are often distinguishable by their abnormal location on clinical examination. Differentiating ectopic from true undescended testes may be difficult if ectopic testes are situated near the inguinal canal, and imaging or surgical exploration may be necessary [2,3,5,47].

In cases where the testis is nonpalpable unilaterally and absent on examination, the differential diagnosis includes true undescended testis (intra-abdominal or canalicular), testicular agenesis (anorchia), ectopic testis, and intrauterine vascular compromise leading to testicular regression [54]. Surgical exploration remains the definitive diagnostic modality, as it allows direct visualization and assessment of testicular tissue, vas deferens, and spermatic vessels. During exploration, the presence of a normal testis, vas deferens, and vessels along the normal descent pathway suggests a true undescended testis. Complete absence of testicular tissue, vas deferens, and vessels indicates testicular agenesis. Residual testicular tissue or remnants support a diagnosis of intrauterine vascular insult or regression, while ectopic testes are identified in aberrant locations, often requiring surgical repositioning or removal if nonviable [6,68]. A retrospective series involving 447 nonpalpable testes demonstrated that approximately 41% were atrophic or absent at surgery; 20% were intra-abdominal, 30% intracanalicular, and the remainder located ectopically, such as suprascrotal, superficial inguinal pouch, or perineal positions [39].

In phenotypic males presenting with a nonpalpable testis and hypospadias, evaluation should include assessment for disorders of sex development (DSDs), such as mixed gonadal dysgenesis or ovotesticular DSD, which may influence management and prognosis [69]. The differential diagnosis in phenotypic males with bilateral nonpalpable testes varies with age. Neonates and infants require immediate evaluation because the differential includes potentially life-threatening DSDs like congenital adrenal hyperplasia (CAH), especially the salt-wasting form caused by 21-hydroxylase deficiency. Although CAH rarely presents with a non-ambiguous male phenotype, early diagnosis via newborn screening and hormonal assessment are essential. The evaluation includes karyotyping to determine chromosomal sex, pelvic ultrasonography to locate gonadal structures and assess for Müllerian derivatives, and hormonal assays such as serum 17-hydroxyprogesterone, testosterone, DHEA, androstenedione, and cortisol to evaluate adrenal function and gonadal activity. Measurement of gonadotropins (LH, FSH) and Müllerian inhibiting substance (MIS) helps assess testicular tissue presence and function, especially during the “mini-puberty” period at around six weeks of age. In cases of suspected CAH, serial electrolyte monitoring and early intervention are vital due to the risk of salt-wasting crises [70,71,72].

In older children, the differential diagnosis includes bilateral testicular absence, bilateral undescended testes, testicular atrophy, and DSDs. The diagnostic approach often involves hormonal testing, imaging, and surgical exploration. Hormonal stimulation tests, such as hCG stimulation, can help confirm the presence of functional testicular tissue; however, current evidence supporting their routine use is limited. The initial assessment should include serum testosterone, gonadotropins, and MIS. Elevated gonadotropins, undetectable MIS, and low baseline testosterone with little or no response to hCG stimulation suggest testicular absence. Nevertheless, normal gonadotropin levels or detectable MIS warrant surgical exploration due to potential complications associated with nonpalpable testicular tissue, including testicular cancer or occult torsion [73,74,75,76,77].

The overall diagnostic approach emphasizes a systematic assessment based on testis palpability, location, associated phenotypic features, and age, with multidisciplinary collaboration among urologists, endocrinologists, and geneticists to optimize diagnosis and management [2,5].

Timely and appropriate referral is crucial for the optimal management of cryptorchidism in phenotypic males. Neonates with bilateral nonpalpable testes or suspected disorders of sex development (DSD) should be referred immediately to a multidisciplinary team comprising pediatric urologists, endocrinologists, and geneticists for comprehensive evaluation and management. Phenotypically male newborns presenting with bilateral nonpalpable testes, or unilateral nonpalpable testes accompanied by hypospadias or other signs suggestive of DSD, require prompt specialist assessment. In older boys beyond infancy, bilateral nonpalpable testes warrant referral to a pediatric urologist for diagnostic laparoscopy or exploratory surgery to locate the testes. Some clinicians may consider hormonal evaluation, such as human chorionic gonadotropin (hCG) stimulation tests, to assess the presence and functionality of testicular tissue before surgical intervention. For infants with a congenital unilateral nonpalpable testis, referral for examination under anesthesia followed by exploratory surgery is recommended, ideally up to 12 months of age, to facilitate early intervention that can improve outcomes and reduce risks related to fertility and malignancy. Palpable undescended testes in infants, whether unilateral or bilateral, should also be referred for evaluation and potential orchiopexy, preferably up to 12 months of age, to promote normal testicular development and minimize complications. In cases where a previously palpable testis is no longer palpable on subsequent examinations, indicating possible ascent, prompt referral for evaluation and orchiopexy is indicated, as early correction can prevent future infertility and malignancy risks. When palpable testicular tissue is present in the scrotum but appears atrophic or hypoplastic, referral for surgical assessment is advised to exclude intra-abdominal testicular tissue, which carries a higher risk of malignancy. Additionally, when clinical examination cannot definitively distinguish between retractile, ectopic, or true undescended testes at any age, referral to a pediatric urologist is essential [2,3,5,12,78,79,80,81].

## 6. Risk of Subfertility and Testicular Cancer

Men with a history of cryptorchidism, including ascending or acquired undescended testes, exhibit a heightened prevalence of impaired spermatogenesis, characterized by reduced sperm counts, decreased sperm quality, and diminished fertility rates compared to men with normally descended testes [82,83]. This association reflects underlying disruptions in testicular development and function, which are likely multifactorial, involving genetic, hormonal, and developmental abnormalities. Notably, some of these impairments may be partially reversible with timely surgical correction. The extent of spermatogenic impairment correlates strongly with prepubertal germ cell counts and the maturation status of testicular cells at the time of orchiopexy [84]. Specifically, germ cell loss and dysfunction tend to increase with bilateral involvement and with longer durations of testes residing in a suprascrotal or intra-abdominal position. Studies suggest that intra-abdominal and intracanalicular testes are similarly affected, likely due to the elevated temperatures outside the scrotum that compromise the spermatogenic environment [85,86].

Endocrinologically, elevated serum follicle-stimulating hormone (FSH) levels and decreased inhibin B concentrations serve as biomarkers indicating Sertoli cell and seminiferous tubule dysfunction [87,88]. Most investigations reveal that, despite impaired spermatogenesis, Leydig cell function—responsible for testosterone production and virilization—remains relatively preserved in men with a history of cryptorchidism [89].

Evidence indicates that early orchiopexy is associated with more favorable testicular histology and better fertility potential [90]. Men who underwent orchiopexy before age two exhibited higher inhibin B levels and lower FSH concentrations compared to those operated on later, suggesting preserved Sertoli cell function and spermatogenic capacity. Conversely, observational data suggest that orchiopexy performed after age one to two years, but before age 12, has a limited impact on long-term fertility outcomes, underscoring the importance of early intervention. Overall, these findings support current recommendations advocating for orchiopexy within the first year of life to optimize fertility prospects [91,92,93,94].

Men with a history of cryptorchidism are also at increased risk of developing testicular germ cell tumors, compared to the general population, where the baseline age-adjusted incidence is approximately 5.4 per 100,000 males. Quantitatively, the magnitude of this increased risk varies across studies, but meta-analyses consistently demonstrate a significant elevation. A 2013 meta-analysis of nine case–control and three cohort studies, encompassing over 2 million boys, reported a pooled odds ratio of 2.9 for testicular cancer among individuals with a history of cryptorchidism [95]. Nevertheless, heterogeneity among studies, potential publication bias, and variable quality limit definitive conclusions. A more recent 2023 meta-analysis, which included surgically treated individuals before adulthood, found that the risk of testicular cancer was approximately fourfold higher in men with a history of cryptorchidism compared to those without [96].

The risk is further amplified in cases of bilateral cryptorchidism, intra-abdominal testes, or associated endocrinological abnormalities or chromosomal anomalies. Additionally, cryptorchidism is a recognized risk factor for germ cell neoplasia in situ (GCNIS), the precursor lesion for testicular germ cell tumors. Surgical correction performed prior to puberty appears to mitigate, but does not eliminate, the risk of testicular cancer. In a large cohort study involving over 16,000 men who underwent orchiopexy between 1964 and 1999, the overall incidence of testicular cancer was 56 cases over a cumulative follow-up of approximately 210,000 person-years [97]. The relative risk was higher in those treated at age 13 or older, with a risk ratio of 5.4, compared to a risk ratio of 2.2 in those treated before age 13, reinforcing the benefit of early surgical intervention. Importantly, surgical correction does not completely eliminate the increased risk, as testicular tumors can still develop in the normally descended contralateral testis. Meta-analyses have demonstrated that approximately 21% of tumors in men with unilateral cryptorchidism occur in the normally descended testis, indicating that factors beyond malposition contribute to tumorigenesis [98,99,100].

## 7. Management

The primary objective in managing undescended testes is to achieve proper testicular positioning within the scrotum, either through orchidopexy (surgical fixation) of viable testes or, in cases of nonviable remnants, via removal of atrophic or nonfunctional tissue [47,101]. Proper scrotal placement of the testes is critical, as it reduces the risk of complications such as testicular torsion and traumatic injury, particularly for intracanalicular testes located along the inguinal canal [102]. Additionally, scrotal positioning facilitates easier clinical examination, which is essential for ongoing surveillance for malignancy and other testicular pathologies [2,3].

Early surgical intervention, ideally before the age of 18 months, has been associated with improved outcomes, including preservation of spermatogenic potential and decreased risks of infertility and testicular malignancy [103]. Furthermore, positioning the testes in a normal, dependent scrotal location may contribute positively to psychosocial development and body image, although the psychological impact has not been extensively studied [104,105].

Surgical correction, specifically orchidopexy, remains the mainstay of treatment for undescended testes and is widely regarded as the definitive management approach. Historically, hormonal therapy—primarily with human chorionic gonadotropin (hCG) or gonadotropin-releasing hormone (GnRH)—has been employed to induce testicular descent. However, evidence indicates that hormonal therapy alone has limited efficacy, with success rates generally below 20–30%, and it does not significantly alter long-term outcomes related to fertility or malignancy risk [106,107]. Current consensus recommends reserving hormonal therapy as an adjunct rather than a primary treatment. Some centers utilize hormonal agents preoperatively to potentially facilitate descent, especially in cases of palpable testes, intending to reduce the extent of surgical intervention needed. Additionally, hormonal therapy may be considered in specific contexts where surgical risks are contraindicated or as part of fertility-enhancement strategies in older boys, although definitive evidence supporting its routine use is lacking [47,108,109,110].

### 7.1. Surgical Treatment

Orchiopexy is the standard surgical procedure for relocating palpable undescended or ectopic testes. The procedure involves mobilization of the testis and spermatic cord, followed by fixation within the scrotum. The surgical approach typically uses inguinal and/or scrotal incisions to preserve the testicular vascular supply and ensure a tension-free placement [111,112]. In cases where the testis appears normal in size and morphology, and the spermatic vessels are of adequate length, primary orchiopexy is feasible. Success rates for primary orchiopexy are high, with a systematic review reporting an average success rate of approximately 96%, defined by the testis residing in the scrotum postoperatively [109]. A comprehensive understanding of retroperitoneal anatomy is essential for mobilizing high intra-abdominal testes safely and effectively, which is best achieved by surgeons with specialized expertise in pediatric urology [113]. Orchiopexy is generally safe. The most significant complication is testicular atrophy, which may result from ischemic injury during dissection of the spermatic vessels or postoperative swelling and inflammation. The reported pooled incidence of testicular atrophy is approximately 1.8%. Other potential complications include testicular reascent, inguinal hernia, infection, and bleeding [114,115,116]. Postoperative management involves activity restrictions to prevent testicular displacement. Children are advised to avoid straddle toys (such as bicycles) for several weeks. Sports activities are typically limited in older children to minimize trauma risk. Routine postoperative examinations are recommended at one to two weeks to assess wound healing and testicular position, with a more comprehensive evaluation at three months to confirm testicular size and location [2,5].

Non-palpable testes present a unique clinical challenge, as their location and viability are not immediately apparent through physical examination alone. The gold standard for diagnosing and managing non-palpable testes remains surgical exploration [63]. The primary goals of surgery are to determine whether the testis is present, assess its viability, and perform orchiopexy if feasible. During exploration, approximately 10% of cases reveal blind-ending spermatic vessels, indicating that the testis is absent, either due to intrauterine regression or previous atrophy [117,118]. There are two main surgical approaches: an open inguinal approach and a laparoscopic approach. The choice depends on the surgeon’s expertise and the specific clinical scenario. Examination under anesthesia can sometimes provide additional information, as some testes that are non-palpable in the clinic may become palpable during anesthesia [63]. During this assessment, the surgeon may detect a small nubbin or remnant in the inguinal canal or scrotum, which can be biopsied or excised. Histological examination of such remnants often shows atrophic tissue, hemosiderin deposits, and atretic vasculature, confirming prior testicular regression. Laparoscopy has become the preferred minimally invasive technique for intra-abdominal testes, as it allows direct visualization of intra-abdominal structures, assessment of the spermatic vessels, vas deferens, and the presence or absence of testicular tissue. If an intra-abdominal testis is identified and deemed viable, laparoscopic orchiopexy can be performed to mobilize and fix the testis within the scrotum. Conversely, if the testis is nonviable or absent, the procedure may be terminated, and the remnant tissue or atrophic testis can be removed to reduce the risk of future complications [119,120,121,122].

In cases where an inguinal hernia coexists with an undescended testis, prompt repair of the hernia at the time of orchiopexy is indicated, especially if incarcerated or strangulated. If no hernia is present, prophylactic hernia repair can be performed during orchiopexy to prevent future herniation [123].

### 7.2. Hormonal Treatment

Currently, hormonal therapy is generally not recommended for the management of undescended testes. However, historically, some clinical centers have employed hormonal treatments to induce testicular descent, often as an adjunct to surgical correction to potentially improve fertility outcomes. Testicular descent depends heavily on the local concentration of testosterone within the inguinoscrotal region, which systemic administration typically cannot achieve. Gonadotropin-based therapies, such as urine-derived human chorionic gonadotropin (hCG) or gonadotropin-releasing hormone (GnRH) analogs, have been used to stimulate Leydig cells within the testes to produce endogenous testosterone, thereby increasing local testosterone levels and facilitating descent [124,125,126].

A 2012 systematic review that included seven heterogeneous studies involving 995 boys and 1215 testes found that hormonal treatment was associated with a modest increase—around 10%—in rates of testicular descent compared to placebo. The success rates varied widely across individual studies, ranging from 9% to 62%. The initial position of the testis influences the likelihood of success, with more distal (glandular or inguinal) testes generally responding better than proximally located (abdominal) testes. It is also important to consider that some testes classified as undescended may actually be retractile, which are more likely to respond to hormonal therapy. Evidence suggests that hormonal treatment appears effective mainly for retractile testes rather than truly undescended testes [66,109,127].

There is limited data on the long-term effects of hormonal therapy, including impacts on testicular cancer risk or fertility. Furthermore, no clear evidence indicates the superiority of any specific hormonal agent or dosing regimen. Both GnRH analogs and urine-derived hCG have shown similar efficacy in achieving testicular descent, with success rates reported between 6% and 35%. Adverse effects are generally mild and transient, typically resolving within six months of treatment. The most commonly reported side effects include signs of virilization—such as increased penile size, body hair growth, and erections—and behavioral changes like increased aggressiveness [2,128,129].

## 8. Guidelines

### 8.1. American Urological Association

Providers should obtain a detailed gestational history during the initial evaluation of boys suspected of having cryptorchidism, as this information is crucial for guiding management. At each recommended well-child visit, primary care providers should palpate the testes to assess their quality and position, ensuring early detection of any abnormalities. If a boy has a history of cryptorchidism detected at birth and the testes have not descended spontaneously by six months corrected for gestational age, providers should promptly refer the infant to an appropriate surgical specialist for timely evaluation. Similarly, boys with the possibility of newly diagnosed (acquired) cryptorchidism after six months, corrected for gestational age, should also be referred for specialist assessment.

In cases of phenotypic male newborns presenting with bilateral nonpalpable testes, providers must immediately consult a specialist to evaluate for a potential disorder of sex development (DSD), as this is a critical step. Before referral, ultrasound or other imaging modalities are generally not recommended in the evaluation of boys with cryptorchidism, since these studies rarely influence decision-making. When assessing for DSD, providers should consider the severity of hypospadias in conjunction with cryptorchidism, as increasing severity may indicate an underlying disorder.

For boys with bilateral, nonpalpable testes who do not have congenital adrenal hyperplasia (CAH), measuring Müllerian Inhibiting Substance (MIS or Anti-Müllerian Hormone [AMH]) levels, along with additional hormone testing if indicated, can help evaluate for anorchia. In boys with retractile testes, clinicians should monitor testicular position at least annually to detect secondary ascent. Hormonal therapy to induce testicular descent is not recommended, given the low response rates and lack of evidence supporting long-term efficacy.

If spontaneous testicular descent has not occurred by six months corrected for gestational age, surgical intervention should be performed within the following year. For prepubertal boys with palpable cryptorchid testes, surgical specialists should carry out scrotal or inguinal orchidopexy. In cases with nonpalpable testes, examination under anesthesia is recommended to reassess palpability; if testes remain nonpalpable, surgical exploration with potential abdominal orchidopexy should be undertaken. During surgical exploration for nonpalpable testes, the surgeon should identify the status of the testicular vessels to guide subsequent management.

In boys with a normal contralateral testis, surgical specialists may perform an orchiectomy of the undescended testis if the testis is very short, dysmorphic, hypoplastic, or if the boy is postpubertal, especially when testicular vessels and vas deferens are very short. Lastly, it is essential to counsel boys with a history of cryptorchidism or monorchidism and their parents about potential long-term risks, including infertility and cancer, providing education and reassurance to support informed decision-making and follow-up [78].

### 8.2. Canadian Urological Association

Physical examination is the cornerstone of cryptorchidism evaluation and should be conducted by an experienced healthcare provider in a warm, relaxed environment. Documentation of this exam should include the patient’s history, noting factors such as prematurity, scrotal asymmetry, whether the gonad(s) is palpable, and any associated genitourinary abnormalities like hypospadias. In phenotypic males with bilateral non-palpable gonads, there should be a high index of suspicion for congenital adrenal hyperplasia with a 46XX karyotype, along with other disorders of sexual development; appropriate workup should be performed before discharge to rule out salt-wasting conditions.

If cryptorchidism is identified on a newborn exam, regular monitoring is warranted to assess for spontaneous descent, and a referral for specialized evaluation should be made at or before six months of corrected age. Imaging studies such as ultrasound, computed tomography, or magnetic resonance imaging are generally unnecessary, expensive, potentially misleading, and not routinely indicated. They may be selectively ordered after specialist evaluation, particularly in cases suspected of disorders of sexual development or prior to surgical intervention, at the discretion of the specialist.

In children diagnosed with cryptorchidism past six months of corrected age, conservative management (expectant approach) generally has no role unless the child has significant comorbidities or high anesthetic risk. Children with retractile testicles should be examined regularly, and the location of the gonad should be clearly documented, especially in the absence of an active cremasteric reflex. If the testicle is observed to ascend into an ectopic or undescended position, specialist referral is warranted.

Acute abdominal or inguinal pain in a child with cryptorchidism should prompt consideration of testicular torsion, requiring urgent surgical assessment. A genital exam should be performed and documented in all boys presenting with such pain, indicating the presence and location of the testicles.

Hormonal therapy’s impact on subsequent gonadal function remains uncertain, and it offers no advantage over timely surgical correction. There is no indication for medical or surgical intervention in children with retractile testicles. For palpable undescended testicles, surgical approaches—either prescrotal or inguinal—should be chosen based on the gonad’s location, the ability to manipulate it into the scrotum, and the surgeon’s preference and expertise.

If the testicle is not palpable during preoperative physical evaluation, an exam under anesthesia should be performed at the start of surgical exploration. In approximately 10–15% of cases, the gonad may become palpable during this exam, allowing for a tailored surgical approach. The goal of orchidopexy is to position the gonad in its normal anatomical location, which should be confirmed and documented during postoperative follow-up. Surgical procedures should also address any associated abnormalities, such as a patent processus vaginalis or inguinal hernia.

The role of contralateral prophylactic orchidopexy in unilateral cryptorchidism or monorchidism to prevent future testicular torsion remains controversial. This decision should be discussed with the family, highlighting the rationale and potential risks, and families should be warned about the importance of urgent evaluation if acute testicular pain occurs.

The diagnosis of an absent, vanishing, or atrophic testicle is established through surgical exploration, with findings such as a blind-ending vas deferens and vessels, absence of the testicle or nubbin, and pathological examination (including hemosiderin, testicular tissue, vas deferens, and vessels) clearly documented to prevent future concerns and re-assessment needs.

All patients should receive education on regular testicular self-exam following orchidopexy and be advised to seek medical attention if abnormalities are palpated or if there is a sudden increase in testicular size. Patients with delayed puberty should be referred for endocrine assessment, and an infertility specialist should evaluate those with concerns about future fertility. This is particularly important for boys at high risk of hormonal or fertility issues, such as those with bilateral intra-abdominal testicles, cryptorchidism in a solitary gonad, or atrophy after attempted orchidopexy [130].

### 8.3. European Association of Urology

For boys with retractile testes, do not offer medical or surgical treatment; instead, closely follow them up until puberty. If a boy presents with an undescended testis, it is recommended to offer surgical orchidolysis and orchidopexy before the age of 12 months, and at the latest by 18 months.

In neonatal males with bilateral non-palpable testes, it is important to evaluate for possible disorders of sex development (DSDs). In cases where non-palpable testes are present without evidence of DSDs, a laparoscopic intervention should be offered due to its high sensitivity and specificity in identifying intra-abdominal testes, allowing for potential treatment during the same session.

Routine hormonal therapy is not generally recommended for testicular descent, whether as adjuvant or neo-adjuvant treatment. Instead, each patient should be evaluated on an individual basis to determine the appropriate management.

In cases of bilateral undescended testes, endocrine treatment may be considered to potentially enhance future fertility. For post-pubertal boys or older individuals with an undescended testis and a normal contralateral testis, the option of removal should be discussed with the patient or parents because of the theoretical risk of malignancy later in life [124].

### 8.4. Comparison of International Guidelines

The three guidelines—American Urological Association (AUA), Canadian Urological Association (CUA), and European Association of Urology (EAU)—present largely aligned principles regarding the evaluation and management of cryptorchidism but differ in specific recommendations and emphasis, reflecting regional practices and interpretations of the evidence [78,124,130].

All three guidelines agree that physical examination remains the cornerstone of diagnosis, emphasizing that it should be performed by experienced providers in a warm, relaxed environment. The AUA and CUA stress the importance of documenting the patient’s history, including factors such as prematurity, scrotal asymmetry, gonad palpability, and associated genitourinary abnormalities, with the CUA specifically highlighting the high suspicion of congenital adrenal hyperplasia in cases of bilateral nonpalpable gonads, necessitating appropriate workup before discharge. Similarly, the EAU emphasizes careful evaluation for DSDs in cases of bilateral non-palpable testes, recommending laparoscopic intervention in the absence of evidence for DSDs, due to its high sensitivity and specificity in identifying intra-abdominal testes. The AUA and CUA concur that ultrasound and other imaging modalities are generally unnecessary in initial assessment, citing their limited influence on management decisions, but the CUA notes that imaging may be selectively ordered after specialist evaluation, especially when considering surgery or suspected DSDs.

Regarding the timing of intervention, all guidelines advocate for early management. The AUA recommends prompt referral for surgical evaluation if spontaneous descent has not occurred by six months corrected age, with orchidopexy ideally performed within the following year. The EAU emphasizes offering surgical orchidolysis and orchidopexy before 12 months of age, and at the latest by 18 months. The CUA suggests that in children diagnosed past six months of corrected age, expectant management has little role unless significant comorbidities or high anesthetic risk are present, highlighting regional differences in management philosophy.

The treatment approach for retractile testes is consistent across all three, with no surgical or medical intervention recommended; instead, close monitoring until puberty is advised. The CUA emphasizes regular examination and documentation of gonad position, especially if ascent occurs. The guidelines also agree that hormonal therapy to induce descent is not recommended, citing low response rates and lack of evidence supporting long-term benefit; the EAU and AUA explicitly state that hormonal therapy offers no advantage over timely surgical correction.

In cases of non-palpable testes, all three guidelines recommend laparoscopic exploration as the preferred diagnostic and therapeutic approach, given its high sensitivity and ability to allow concurrent treatment. The AUA and CUA specify that during surgical exploration, the status of the testicular vessels should be identified to guide subsequent management. The CUA additionally emphasizes the importance of evaluating for disorders of sex development before surgical intervention, especially in bilateral non-palpable cases, to exclude conditions like CAH.

Finally, regarding malignancy risk, the EAU recommends discussing testicular removal in post-pubertal cases with normal contralateral testes due to potential malignancy risk, whereas the AUA and CUA highlight the importance of counseling boys with a history of cryptorchidism about lifelong risks, including infertility and cancer, and the need for ongoing surveillance.

## 9. Future Directions

Future research in the field of cryptorchidism should focus on several key areas to enhance understanding and improve patient outcomes. First, elucidating the precise molecular and genetic mechanisms underlying testicular descent and the etiology of cryptorchidism remains a priority. Advanced genomic studies, including whole-exome and genome-wide association studies, could identify novel genetic variants and pathways involved in testicular migration failure, potentially leading to targeted therapies or early predictive markers. Second, there is a need for standardized, longitudinal studies to better define the natural history of retractile and acquired cryptorchidism, as well as the long-term effects of various management strategies on fertility and oncological risks. Third, improving diagnostic modalities—such as developing highly sensitive, non-invasive imaging techniques or biomarkers—could refine the localization and viability assessment of nonpalpable testes, reducing reliance on surgical exploration and enabling more precise, individualized treatment plans. Fourth, further investigation into the role of hormonal therapies, including optimal timing, dosing, and patient selection, is warranted to clarify whether specific subsets of patients could benefit from adjunct hormonal treatment, especially in cases where surgery poses increased risks. Finally, researchers should also explore the psychosocial and quality-of-life impacts of cryptorchidism and its treatment, incorporating patient-centered outcomes and addressing potential disparities in care across different populations. Collectively, these future directions aim to deepen our understanding of cryptorchidism’s pathogenesis, optimize diagnostic accuracy, and refine management protocols to improve long-term reproductive and oncological health in affected individuals.

## 10. Conclusions

Based on the current evidence, early diagnosis and timely surgical intervention, preferably before 12 to 18 months of age, are crucial for optimizing outcomes in children with cryptorchidism. Orchidopexy effectively reduces the risks of infertility, testicular torsion, trauma, and malignancy, while hormonal therapy has limited efficacy and is generally not recommended as a primary treatment. Ongoing surveillance and management are essential to address potential long-term complications, including subfertility and increased testicular cancer risk, emphasizing the importance of a multidisciplinary approach for personalized care.

## Data Availability

No new data were created or analyzed in this study.

## Abbreviations

The following abbreviations are used in this manuscript:

DSD	Disorders of Sex Development
GCNIS	Germ Cell Neoplasia in Situ
INSL3	Insulin-Like Factor 3
MIS	Müllerian Inhibiting Substance
MRI	Magnetic Resonance Imaging
hCG	Human Chorionic Gonadotropin
GnRH	Gonadotropin-Releasing Hormone
CAH	Congenital Adrenal Hyperplasia
FSH	Follicle-Stimulating Hormone
LH	Luteinizing Hormone
AMH	Anti-Müllerian Hormone
DHEA	Dehydroepiandrosterone
AUA	American Urological Association
CUA	Canadian Urological Association
EAU	European Association of Urology
